# A Successful Case of Resection of the Bowels

**Published:** 1881-09

**Authors:** 

**Affiliations:** Prof. Treudelenburg’s Clinic in Rostock


### Translations.

---

A Successful Case of Resection of the Bowels.—Reported by Dr. Roggenbon from Prof. Treudelenburg's Clinic in Rostock.

Translated from the German.

The operation ot resection of the bowels has formerly been considered as a very bold undertaking, partly on account of the difficulty of uniting the ends of the bowels so perfectly that no fecal matter can come out through the wound (on the suture-holes), while at the same time the lumen of the bowels must be maintained in its size, partly on account of the danger of peritonitis. Although the first mentioned difficulty may be considered overcome since Lembert invented his suture, resection of the bowels can only be counted among rational operations, since we have learned to prevent septic peritonitis by the use of antiseptic precautions. We see, therefore, that this operation, which formerly was performed very exceptionally, has suddenly become recognized as justifiable in the case of malignant newgrowths on the bowels, and commences in cases of gangrenous herniæ to rival the operation for formation of artificial anus.

But nobody doubts that this operation first will gain its greatest practical value, when we, in cases of strangulated hernia, in which the bowels have suffered considerably by the incarceration, but are not distinctly gangrenous, have the choice between resection and reposition of a suspicious hernia. The cause of death after herniotomy is, according to the author, in the majority of cases secondary gangrene of the bowels, or peritonitis, on account of lesions of the bowels.

Considering the results of resection of the bowels since Czerny's paper on the subject, in 1877, the author would think the operation advisable in cases in which the bowels were found in a state of intense congestion. To draw the line distinctly, is difficult. We might, in such cases, let the bowels remain out for a time (as Graefe has advocated in *Berliner Klinische Wochenschrift*, No. 8, 1881), although the chance of a successful operation surely would not improve by it.

The author thereafter reports a case of resection. The patient, a lady, 74 years of age, entered the hospital February 8th, 1881, on account of a strangulated femoral hernia on the right side. She had suffered from rupture for 30 years, but had kept it back with a truss. She had been treated in the hospital several times for gall-stone colic. Eight days previously she had again an attack of colic, combined with icterus. The day before she entered the hospital the rupture came out during difficult defecation, but was brought back again. In the evening it came out again, and soon became strangulated. Taxis was not successful, and permatony was, therefore, performed 24 hours after the accident.

The bowels had a dark-blue color, with many ecchymoses in the walls, but no gangrene was discovered. It was decided to reposit the bowels, partly on account of the age of the patient; but during this act, which was rather difficult, the bowels tore next to the mesentery. The canal was immediately compressed so that none of the fluid entered the peritoneal cavity, and it was decided to resect the strangulated part of the bowels. The section was done in the healthy part of the bowels and a piece, 32 centimeters long, removed with a wedge of the mesentery. The sutures were applied first to the part of the bowels adjacent to the mesentery (as it is difficult to secure a good union here, if we apply the sutures to the mesentery first); Czerny's modification of Lembert's suture was used, which consists of two rows of sutures, of which the first only passes through the serous covering of the bowels, next to the wound, while the second row consists of Lembert's sutures, applied alternately with the first ones. Eleven and ten sutures were applied in the different rows, and fine silk, dipped in a 4 per cent. solution of carbolic acid, used.

The canal having been thoroughly incised to prevent any tearing of the sutures, the reposition was effected. The sac and omentum were ligated and cut off; the wound was closed with five sutures and a drain tube left in the lower angle. Recovery took place without any unfavorable symptoms. The bowels moved on the fifth day after using castor oil. In the course of eight days a circumscribed tumor appeared in the ileo-cœcal region, which disappeared slowly from pressure under symptoms of borborygmi. The wound in the bowels united by first intention, which does not always take place. In a case of resection by Polano, fœcal discharge took place from the wound on the ninth day; in a case reported by Dittel, on the fifth day; in one by Kacher and two by Hageborn, respectively on the eighth and fifth days, but all five cases recovered at last. As these cases show that gangrene may occur of the wound, the author advises not to tie the sutures of the second row too firmly. To close the abdominal cavities during the operation and prevent entrance of fœcal matter, compression with a sponge over the canal showed itself to be amply sufficient.—*Berliner Klinische Wochenschrift*, July 18, 1881.

